# Integrated Metabolomics and Transcriptomics Analyses Reveal the Candidate Genes Regulating the Meat Quality Change by Castration in Yudong Black Goats (*Capra hircus*)

**DOI:** 10.3390/genes15010043

**Published:** 2023-12-27

**Authors:** Songjian Yang, Xinyue Zhang, Xingchun Li, Jikang Zheng, Le Zhao, Chengli Fan, Yongju Zhao

**Affiliations:** 1 Chongqing Key Laboratory of Forage & Herbivore, Chongqing 400715, China; ysj018882023@163.com (S.Y.); xyxy662303@163.com (X.Z.); lxc500112@163.com (X.L.); zjk990607@163.com (J.Z.); zhaole0228@163.com (L.Z.); chenglifan@126.com (C.F.); 2 Chongqing Key Laboratory of Herbivore Science, Chongqing 400715, China; 3 College of Animal Science and Technology, Southwest University, Chongqing 400715, China

**Keywords:** goat, meat, metabolomics, transcriptomics, castration

## Abstract

Yudong black goats (YDGs) are a local breed in southwest China that possess unique meat qualities and produce a high meat yield, making them ideal models for studying goat meat quality. Castration may decrease off-odors, significantly change metabolites and improve meat quality. Using multi-omics techniques, this study focused on Yudong black goat wethers (YDW, n = 4) and Yudong black bucks (YDB, n = 4). The findings revealed that 33 differentially expressed genes (DEGs) and 279 significantly changed metabolites (SCMs) influenced goat meat quality by affecting fat accumulation and lipolysis regulatory processes. Herein, several candidate genes (*IGF1*, *TNNT2*, *PPP2R2C*, *MAPK10* and *VNN1*, etc.) were identified that play a role in regulating meat quality, non-castrated and castrated, alongside a series of metabolites that may serve as potential meat quality biomarkers. Lipids (triglycerides, oxidized lipids_5-iso PGF2VI, ceramide (t18:1/36:2(2OH)) and Carnitine C20:5, etc.) were significantly higher in the castrated goats. These results revealed that lipids and hydrophilic metabolites were affected by castration, which might be beneficial in terms of goat meat quality. This study aimed to investigate the differences in meat quality between uncastrated and castrated male goats and the possible molecular regulatory mechanisms.

## 1. Introduction

China, being a vast country with a long history of goat farming, possesses abundant goat breeding resources. Among consumers, goat meat is highly sought after due to its low fat content and high protein content, which offer significant health benefits, as well as for its unique flavor. With the continuous improvement in living standards, ensuring the availability of high-quality meat has become a primary objective in animal breeding [1]. Meat quality is comprehensively assessed by a series of indicators, such as meat color, intramuscular fat content (IMF), pH value, water holding capacity, cooking loss and fatty acid content. YDGs, one prominent local goat breed in China originating from the southwest region, is renowned for their rapid growth and high-yielding meat production performance. However, an excessive variation in meat quality will detrimentally affect sales and hinder the growth of the YDG meat industry.

Goat meat quality is influenced by various factors, which can be divided into non-genetic and genetic factors. Non-genetic factors, such as the external environment, feeding management and slaughtering methods, vary considerably across the industry [2]. Conversely, genetic factors, including the breed, individual characteristics and tissue parts, exhibit less variability [3,4]. Castration contributes to improved meat quality and makes male goats easier to manage. Moreover, the castration of rams reduces the smell of mutton, suggesting that castration confers some improvement to the quality of the meat [5]. Castration is an important technical means by which the quality of goat meat can be improved [6]. However, how castration affects goat meat quality has yet to be scientifically determined. Little research has been conducted into the effect of castration on meat quality in YDGs. Therefore, it has become particularly important to determine the potential molecular mechanisms by which castration leads to changes in meat quality in goats. These advancements are crucial for enhancing productivity and meeting the demands of the goat meat industry.

In recent years, histological techniques have undergone significant advancements and are now widely utilized to study meat quality [7]. Conversely, transcriptomic analysis can provide comprehensive insights into the genetic mechanisms that underpin meat quality. These data can be coupled with metabolomic analyses, which can offer supplementary information regarding the metabolic changes that occur because of posttranscriptional regulation, thereby shedding light on the biochemical processes and alterations that take place in living systems. For instance, Pan et al. [8] conducted transcriptome sequencing of the longest back muscle in Luchuan and Duroc pigs, revealing pathways involved in skeletal muscle growth, fatty acid metabolism and intramuscular fat (IMF) deposition. Wu et al. [9] performed a comprehensive analysis of the entire transcriptome profiles of the oxidizing biceps femoris and glycolytic external abdominal obliques in yaks, highlighting the differences in metabolic properties between oxidizing and glycolytic muscles. In addition, they constructed regulatory networks associated with muscle fiber type conversion and mitochondrial energy metabolism. Zhang et al. [10] investigated the relationship between dietary energy consumption and meat quality in Tibetan black sheep using metabolomics and Pearson’s correlation analysis. Finally, Liu et al. [11] employed an untargeted metabolomics approach to uncover the changes in small molecules during long-term storage of duck meat. These studies collectively contribute to a deeper understanding of the mechanisms that regulate meat quality through the application of multi-omics analysis techniques.

To cater for the increasing expectation of superior meat quality among consumers, this study aimed to investigate the molecular mechanisms underlying the diversity that regulate goat meat quality by integrating and analyzing multi-omics data.

## 2. Materials and Methods

### 2.1. Animals and Diets 

All experimental procedures involving animals were conducted in accordance with the guidelines and regulations set by the Animal Protection and Utilization Committee of Southwestern University (IACUC-20230227-01). A total of 8 healthy 12-month-old Yudong black goat wethers (YDW, n = 4) (castration at 4 months of age) and Yudong black bucks (YDB, n = 4) were selected as subjects for this study. These animals were reared under similar conditions to ensure consistency. The diets were equally divided into two parts, with feeding at 7:00 a.m. and 7:00 p.m. every day, and freshwater was provided ad libitum. Pangola hay and alfalfa pellets were fed first alongside a mixture of concentrate. The experimental animals were sourced from Chongqing Taifeng Livestock and Poultry Breeding Company Limited (Chongqing, China). 

### 2.2. Measurements, Sample Collection and Chemical Analysis

In this analysis, the carcass weight, carcass length and thoracic depth were recorded and measured immediately after slaughter. The live weight was measured before the goats were sacrificed; the hot carcass weight was obtained within 30–40 min after slaughter. The carcass weight is defined as the weight of the goat with blood, skin, head, hooves, tail and viscera removed. The dressing percentage is the ratio of the hot carcass weight to live weight. For the metabonomic analysis and RNA sequencing, 2 g samples of the longissimus dorsi muscle were collected using a 2 mL RNA-free freezing tube and immediately placed in liquid nitrogen for preservation before being stored at −80 °C until analysis. The longissimus dorsi muscle samples (approx. 20 g) were oven-dried to constant weight and ground to powder to extract IMF. The IMF content was determined using the Soxhlet method (GB 5009.6-2016). The fatty acid content was determined by gas chromatography in accordance with the method for the determination of fatty acids in food (GB 5009.168-2016). The amino acid content was determined using an automatic amino acid analyzer with reference to the determination of amino acids in foodstuffs (GB 5009.124-2016).

The longissimus dorsi muscle samples (approx. 20 g) were kept at 4 °C for 24 h for further meat quality analysis. At 50 min postmortem, the pH of the longest back muscle was determined using a pH meter tipped with a glass electrode. After 30 min of blooming, the meat color on the cut surface of the longest back muscle was measured using a colorimeter (CR-400, Konica Minola, Japan) to determine L*, a* and b* (lightness, redness and yellowness, respectively) in triplicate. The longest back muscle samples (approx. 100 g) were placed in airtight plastic bags and cooked for 35 min in the same water bath. After cooking, the samples were cooled with running water for 30 min, and the surface water was absorbed with filter paper and dried. Weighing recorded before (W1, g) and after (W2, g) cooking was used to calculate the cooking loss ((W1 − W2)/W1 × 100%). Then, each block of meat was cut into 6 rectangular cores (cross-sectional area of 1 cm^2^) parallel to the longitudinal direction of the muscle fibers, and the shear force was measured using a tenderness meter (C-LM3B, Northeast Agricultural University, Harbin, China).

### 2.3. Metabolite Extraction and Metabolomic Analysis

The samples (longissimus dorsi muscle) were retrieved from the −80 °C freezer and thawed on ice. Once thawed, 20 mg of each sample was accurately weighed and transferred into a numbered 2 mL centrifuge tube. A steel ball was added to each tube using forceps, and the samples were homogenized using a ball mill at a frequency of 30 Hz for 20 s. Subsequently, the samples were centrifuged at 3000 rpm for 30 s at 4 °C to sediment the contents at the bottom of the tube (the homogenization time could be adjusted as required). 

Next, 1 mL of lipid extract (methyl tert-butyl ether: methanol = 3:1, *V*/*V*), which contained the internal standard, was added to each tube. The mixture was vortexed for 15 min, which was followed by the addition of 200 μL of water. After vortexing for an additional minute, the tubes were centrifuged at 12,000 r/min for 10 min at 4 °C. Subsequently, 200 μL of the supernatant was carefully pipetted and transferred to a new centrifuge tube and the liquid supernatant was fully evaporated, after which 200 μL of a lipid complex solution (acetonitrile: isopropanol = 1:1, *V*/*V*) was added. The solution was vortexed for 3 min and centrifuged at 12,000 r/min for 3 min. Finally, the supernatant was collected for LC-MS/MS analysis. The chromatographic mass spectra were acquired using ultra performance liquid chromatography (UPLC) coupled with tandem mass spectrometry (MS).

Using the Metware Database (MWDB), which is a self-constructed database of Wuhan Metware Co., Ltd., Wuhan, China, the relevant information was subjected to qualitative analysis based on the retention time (RT) of lipids and their corresponding daughter ions. Then, the obtained lipid mass spectrometry data for the different samples were integrated by calculating the peak area. Furthermore, corrections were made for the integration of the mass spectrometry peaks that corresponded to the same lipid but were found in different samples.

The mass spectrometry data were processed using the Analyst 1.6.3 software, and Pearson’s correlation analysis was performed to examine the lipids in the samples. Lipid differences between groups were then analyzed, and the reliability of the model was assessed using principal component analysis (PCA) and orthogonal partial least squares discriminant analysis (OPLS-DA). The OPLS-DA model was employed to identify differential lipids between groups, with a criterion of variable importance in projection (VIP) > 1 used as the initial screening threshold. Additionally, a criterion of *p*-value < 0.05 was applied to further refine the selection of differential lipids between groups. Functional annotation and enrichment analysis of the differential lipids were conducted using the KEGG database.

### 2.4. RNA Extraction and Illumina Sequencing

RNA was extracted from the samples using the Trizol method, and the resulting RNA was dissolved by adding 50 μL of DEPC-treated water. The quality and integrity of the extracted RNA were assessed using an Agilent 2100 Bioanalyzer (Agilent Technologies, Santa Clara, CA, USA). Once the RNA samples were confirmed to be of sufficiently high quality, the mRNA was isolated and the cDNA was synthesized. Subsequently, library preparation, including end repair, the addition of A-tail, the addition of sequencing adaptors, purification, PCR amplification, denaturation and cyclization were performed. Once the library was constructed, it was submitted to Maiware Metabolism Co., Ltd. (Wuhan, China) for sequencing.

### 2.5. Sample Relationship Analysis

The raw data were filtered using Fastp [12] to remove reads that primarily contained adaptors. All subsequent analyses were conducted using the clean reads. The reference genome of the domesticated goat (Capra_hircus.ARS1.cdna.all.fa) and its corresponding annotation file (http://ftp.ensembl.org/pub/release-107/fasta/capra_hircus/dna/ accessed on 16 March 2023) were downloaded (https://www.ncbi.nlm.nih.gov/ accessed on 13 May 2023). Indexes were built using HISAT [13], and the clean reads were aligned to the reference genome. The gene matching status was calculated using featureCounts [14], and then the fragments per kilobase of transcript per million fragments mapped (FPKM) was calculated for each gene based on its length.

Differential gene expression analysis between the two groups was carried out using DESeq2 [15,16]. The *p*-values were adjusted using the Benjamini–Hochberg method. Corrected *p*-values and log2Fold change ≥1 were used as thresholds to determine statistically significant differential expression changes. Enrichment analysis based on the hypergeometric test was performed using the genome structure annotation file (capra_hircus.ARS1.107.chr.gff3) for KEGG [17] and Gene Ontology (GO) [18]. Pathways and GO terms that exhibited significant enrichment among the differentially expressed genes, compared to the entire genomic background, were identified.

### 2.6. RNA Extraction and Real-Time Quantitative PCR (RT-qPCR)

Primers specific for 6 genes (*β-actin*, *IGF1*, *TNNT2*, *PPP2R2C*, *VNN1* and *MAPK10*) were designed using the Primer Premier 5 software (Primer Premier 6.25). The specificity of the primers was verified online using the NCBI website (https://www.ncbi.nlm.nih.gov/ accessed on 13 May 2023). The primers used for gene expression quantification can be found in Table S1. For first-strand cDNA synthesis, 1 µg of total RNA was used with a Prime Script^TM^ RT reagent kit containing gDNA Eraser (Tiangen, Beijing, China). Subsequently, qPCR was performed on a real-time fluorescence quantitative PCR instrument (Bio-Rad CFX96, Hercules, CA, USA) using a TB Green^TM^ Premix Ex Taq^TM^ II cDNA synthesis kit. Goat β-actin was included as an internal reference, and the 2^−ΔΔCT^ method was utilized to analyze gene expression by normalizing the relative changes.

### 2.7. Joint Analysis of Transcriptomic and Metabolomic Data

To investigate which regulatory networks are potentially responsible for the diversity of goat meat quality, correlation analysis was conducted using the quantitative values obtained for the genes and metabolites in all samples. Pearson’s correlation coefficients were calculated using the “cor” function in R to evaluate the correlation between genes and metabolites. A threshold of |r| > 0.8 and *p*-values < 0.05 were used to identify strong correlations between differentially expressed genes (DEGs) and significantly changed metabolites (SCMs). The significantly related gene–metabolite combinations were subjected to joint biological annotation using the KEGG database. Specifically, only pathways containing both DEGs and SCMs were selected. Key regulatory genes were annotated to identify pathways associated with the metabolome and transcriptome. The resulting network was visualized using Cystoscape (https://cytoscape.org/ accessed on 16 March 2023), a popular tool for visualizing complex biological networks.

### 2.8. Statistical Analyses

All data were analyzed using the SPSS 23.0 software and presented as mean ± standard error of the mean (mean ± SEM). Statistical significance was assessed using the *t*-test, with a *p*-value < 0.05 being considered statistically significant.

## 3. Results

### 3.1. Slaughter Performance, Carcass Traits and Meat Quality

There were no significant differences (*p* > 0.05) in the carcass performance, carcass characteristics and amino acid content between YDB and YDW (Table 1 and Table 2). Compared with the non-castration group, the a* value of the longest back muscle was significantly increased in the castration group. However, the cooking loss (*p* < 0.05) was significantly lower in the non-castration group than in the castration group (Table 3). Compared with the non-castration group, the pentadecanoic acid content of the longest back muscle was significantly increased in the castration group. However, the contents of trans-9-octadecanoic acid, linoleic acid and polyunsaturated fatty acid (*p* < 0.05) were significantly lower in the castration group than in non-castration group. There were no significant differences (*p* > 0.05) in any of the other fatty acid contents (Table 4).

### 3.2. Metabolic Profiling Analysis

**Figure 1:**
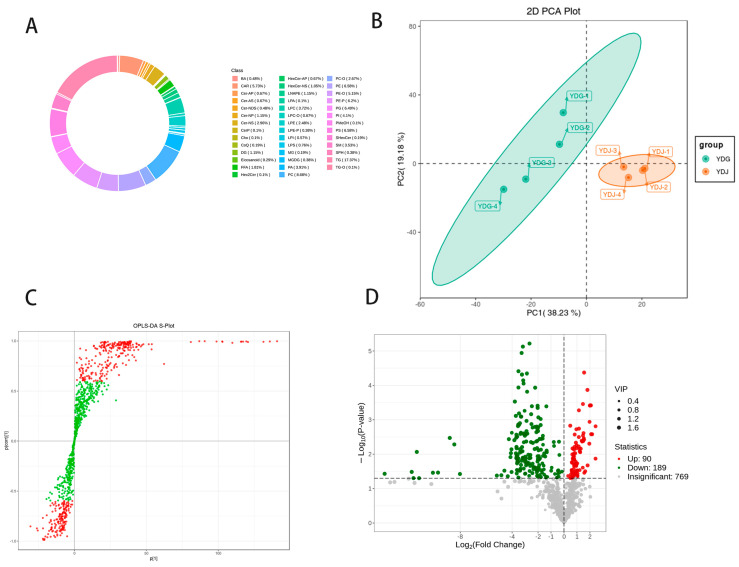

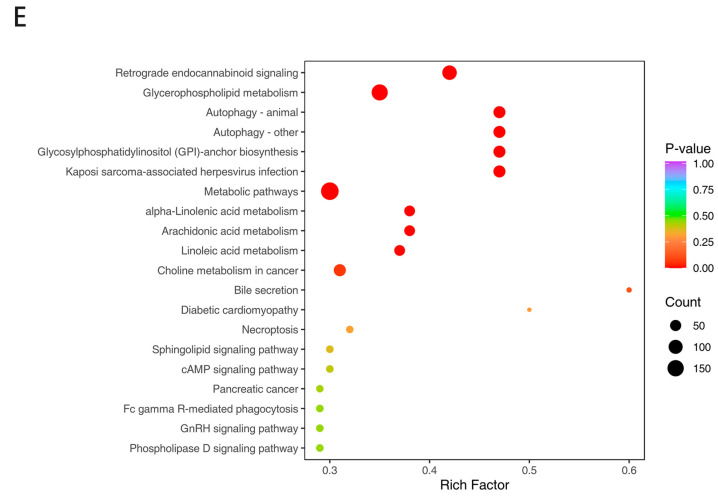
Metabolome data analysis separately in YDB and YDW. (**A**) Percentage distribution of metabolite subclasses. (**B**) Lipid PCA score plot of quality spectrum data of the YDB vs. YDW. (**C**) Score plot of OPLS-DA in the YDB vs. YDW groups. (**D**) Volcano plot illustrating differential lipid abundance in the YDB vs. YDW groups. (**E**) KEGG enrichment maps of differential lipids in YDB vs. YDW groups.

The lipidomic analysis resulted in the detection of 1048 different lipids in all the samples. The major lipid subclasses identified were phosphatidylcholine (PC) at a percentage of 8.68%, phosphatidylethanolamine (PE) at 6.58%, phosphoenolpyruvate acid (PE-P) at 6.2%, phosphatidylglycerol (PG) at 6.49%, phosphatidylserine (PS) at 6.58% and triglyceride (TG) at 17.37% (Figure 1A). To determine the metabolite differences between castrated and non-castrated rams within the same breed, multivariate statistical analysis was performed on the metabolomics data. The principal component analysis revealed significant differences between YDB and YDW (Figure 1B). The lipid group data were further analyzed using the OPLS-DA model, and subgroup scores were plotted to illustrate the differences between the subgroups (Figure 1C). The samples in the YDB vs. YDW comparison groups were clearly distinguishable from each other, indicating significant differences in the metabolite phenotypes between these groups. Regarding metabolite abundance, a total of 279 differential lipids were identified in the lipid group. Among these, there were 189 up-regulated lipids and 90 down-regulated lipids (Figure 1D).

KEGG pathway enrichment analysis was conducted using the differential lipid results. It was found that the differential lipids were significantly enriched in a total of 130 pathways. The top 20 pathways, ranked by their *p*-values, were selected and presented in a scatter plot in ascending order. The results revealed that the different lipids in the YDB and YDW groups showed significant enrichment in 11 metabolic pathways (*p* < 0.05). These pathways included retrograde endocannabinoid signaling, glycerophospholipid metabolism, autophagy-animal, autophagy-other, glycosylphosphatidylinositol (GPI)-anchor biosynthesis, Kaposi sarcoma-associated herpesvirus infection, α-linolenic acid metabolism, arachidonic acid metabolism, linoleic acid metabolism and others (Figure 1E). These findings indicate that the differential lipids identified in the YDB and BJW groups were associated with specific metabolic pathways, highlighting potential regulatory mechanisms underlying the diversity in meat quality.

### 3.3. Transcriptomic Analysis

**Figure 2:**
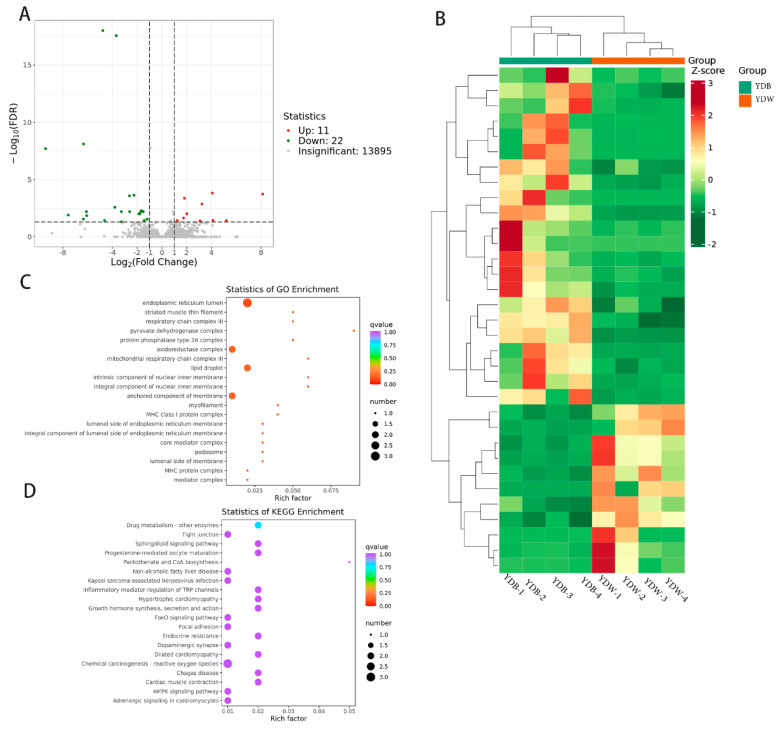
A partial list of bioinformatics analysis results using DEGs. (**A**) Volcano plot displaying differential genes in the YDB vs. YDW groups. (**B**) Hierarchical clustering heatmap of differential genes. (**C**) Scatter plot demonstrating enrichment analysis results in the YDB vs. YDW groups. (**D**) Scatter plot showcasing enrichment analysis results in the YDB vs. YDW groups.

To investigate the regulatory mechanisms that potentially determine goat meat quality, transcriptome sequencing was performed on eight samples of dorsal longissimus muscle tissue. After filtering the raw data, checking the sequencing error rate and GC content distribution, a total of 662, 931, 286 high-quality data points (clean reads) and 149.86 Gb of valid data points (clean bases) were obtained. The valid data for all the samples exceeded 9 Gb, with an error rate of 0.03%, Q20 ≥ 96.87% and Q30 ≥ 90.36%. When the valid data were compared with the goat reference genome, it was found that all pairs had a similarity of ≥95.84% (Table S2). These results indicate that the obtained sequencing data were highly reliable and therefore suitable for subsequent analyses. Pearson’s correlation analysis demonstrated a high correlation (R2 > 0.96) in the FPKM values between the samples. A total of 33 differential genes were identified between the YDB and YDW groups, including 11 up-regulated genes and 22 down-regulated genes (Figure 2A).

Hierarchical clustering analysis was performed on the differential gene sets of the YDB and YDW groups. The data were normalized using Z-scores, and a clustering heat map was generated to visualize the gene expression patterns within each differential grouping. The samples within groups clustered well, displaying similar patterns of differential gene expression (Figure 2B). To gain insights into the functional significance of the differentially expressed genes, GO and KEGG enrichment analyses were conducted for the YDB and YDW groups. The results revealed that the differential genes were enriched in 552 GO entries. These entries were primarily related to functional components such as the endoplasmic reticulum lumen, oxidoreductase complex, lipid droplet and anchored component of the membrane (Figure 2C). These enrichment analyses provide valuable information about the potential functional roles and pathways associated with the differentially expressed genes in the YDB and YDW groups. 

To explore the biochemical metabolic pathways and signaling pathways associated with the DEGs, significant pathway enrichment analysis was conducted using the KEGG database. In the YDB and YDW groups, significant enrichment was identified in pathways such as drug metabolism-other enzymes, progesterone-mediated oocyte maturation, growth hormone synthesis, secretion and action, the AMPK signaling pathway and the adipocytokine signaling pathway (Figure 2D). The differential expression of genes such as *MAPK10*, *IGF1*, *TNNT2*, *PPP2R2C* and *VNN1* was associated with these pathways. These findings reveal the potential involvement of specific metabolic and signaling pathways in the regulation of goat meat quality and provide insights into the molecular mechanisms underlying the observed differences in gene expression between the groups.

### 3.4. Validation of mRNAs by qPCR

**Figure 3:**
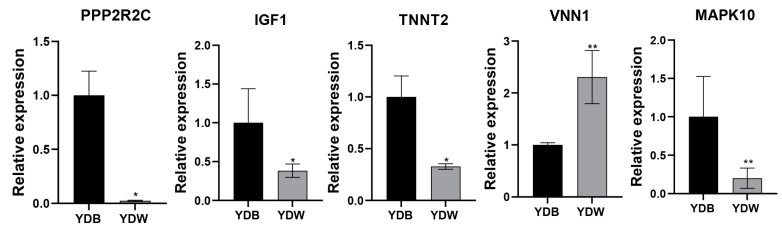
Validation of mRNAs by qPCR. * *p* < 0.05; ** *p* < 0.01.

To validate the expression of selected genes, including *IGF1*, *TNNT2*, *MAPK10*, *PPP2R2C* and *VNN1* in the YDB and YDW groups, RT-PCR analysis was conducted (Figure 3). The results demonstrated significant differences in the expression levels of these genes. Importantly, the variability in expression observed for these five genes was consistent with the RNA-seq data, confirming the reliability and accuracy of the transcriptome sequencing results.

### 3.5. Transcriptomics–Metabolomics Joint Analysis on Meat Quality 

**Figure 4:**
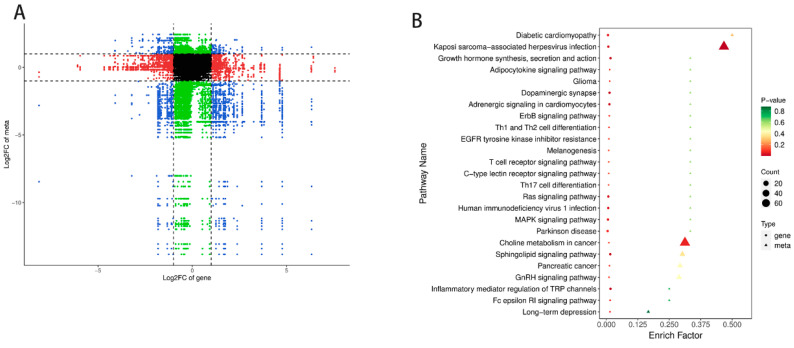
(**A**) Nine-quadrant chart illustrating correlation analysis in the YDB vs. YDW groups. (**B**) Bubble diagrams presenting KEGG enrichment analysis results in the YDB vs. YDW groups.

The regulation of metabolism by an organism involves the precise control of gene expression [19]. To explore additional biological processes affecting meat quality using transcriptomics and metabolomics analyses, correlation tests and joint biological annotation of these changes were performed. The correlation analysis using the quantitative values obtained for the genes and lipids in all the samples revealed that the differentially expressed genes, including *PPP2R2C*, *IGF1*, *VNN1*, *TNNT2* and *MAPK*, were highly correlated with lipids, including oxidized lipids_5-iso PGF2VI, carnitine C20:5, carnitine C22:5, ceremide (d21:3/38:2(2OH)) and ceramide (t18:1/36:2(2OH)), in the YDW and YDW groups (Figure 4A). The KEGG enrichment analysis of the differentially expressed genes and metabolites revealed that in the YDB and YDW groups, 12 differentially expressed genes and 197 differentially expressed lipids were co-enriched in 44 pathways. The pathways related to the regulation of meat quality and lipid metabolism included growth hormone synthesis, secretion and action, sphingolipid signaling, Rap1 signaling, Ras signaling, adipocytokine signaling, MAPK signaling, HIF-1 signaling, cAMP signaling and metabolic pathways (Figure 4B). These findings suggest that castration may have an impact on goat meat quality, and the regulation of meat quality involves complex metabolic and signaling pathways.

## 4. Discussion

Castration is one of the technical tools that are commonly used in the goat industry to facilitate the improvement in the quality of the meat. Rajkumar, V et al. [20] studied the effect of castration and the age of slaughtering on the carcass characteristics, color and fatty acid profile of Barbari goat meat and found that the meat quality and fatty acid content were significantly affected by the age of slaughtering and castration, which is also consistent with the present experimental findings. On the other hand, the castration of goats in general led to an increase in the deposition of fat in the body [21]. However, in the study by Tahir [22], no significant effect of castration on fat deposition was observed in goats. These differences may be related to the rearing conditions and age at castration. In addition, there was no significant effect of castration on the amino acid content of goat meat. This was probably due to the similarity in the protein content of the samples.

Transcriptomics and metabolomics analyses were performed based on transcriptome sequencing and LC-MS/MS systems to explore the complex biological processes affecting meat color phenotypes and other goat meat quality traits. In this study, a broadly-targeted metabolomics approach was employed to investigate the impact of castration on lipid metabolism in goats. A total of 279 differential lipids were identified, and several of these lipids have been found to be associated with the quality of meat. For instance, phosphatidic acid (PA) is known to regulate cellular functions by directly interacting with effector proteins [23] and can promote the activity of mTOR, a key enzyme involved in muscle synthesis and muscle anabolism. Additionally, glycerophospholipid metabolism has been linked to muscle flavor and development [24], and PC has the ability to activate peroxisome proliferator-activated receptors (PPARs) directly, thereby regulating the expression of genes involved in lipid metabolism. PC has also been shown to play a role in the hepatic uptake of fatty acids, muscle β-oxidation processes and the enhancement of fatty acid utilization in muscle cells. Additionally, it has been previously reported that a negative correlation exists between carnitine content and body weight gain in various breeds of pig [25]. Phosphatidylinositol has been implicated as an upstream regulator of Hippo signaling pathway activity [26] and may contribute to IMF deposition [27]. These findings highlight the significant impact of castration on meat quality. However, the specific regulatory mechanisms underlying these effects are not yet fully understood. Therefore, exploring the key regulatory genes associated with differential lipids is crucial to understanding the genetic factors influencing goat meat quality.

In this study, several differentially expressed genes were identified, including *MAPK10*, *IGF1*, *PPP2R2C*, *VNN1* and *TNNT2*, which may play important roles in regulating goat meat quality. These genes could serve as candidate genes for the further investigation of meat traits by integrating transcriptome data analysis. In the YDB and YDW groups, the differentially expressed genes *IGF1*, *MAPK* and differential lipid glycerol diester (DG) (16:0_16:0) were co-enriched in pathways related to growth hormone synthesis, secretion and action, metabolic pathways and various signaling pathways. Notably, IGF-1 promotes cell growth and development, participates in the regulation of protein synthesis and expression in vivo and inhibits glycogen and lipolysis [28]. The MAPK signaling pathway affects fat deposition [29], and the down-regulation of IGF1 gene and up-regulation of the *MAPK* gene, along with the up-regulation of DG (16:0_16:0), suggest a potential role of *IGF1* in promoting fat deposition by inhibiting triglyceride synthesis. In addition, it has been shown that a high slaughter weight also leads to a decrease in muscle triglycerides [30]. Furthermore, the *VNN1* gene, considered a target gene of PPARα, a key regulator of fat oxidation [31], was co-enriched with lipoxinA4 in the metabolic pathway. The lower expression of the *VNN1* gene in the YDB group compared to the YDW group, along with the down-regulation of lipoxinA4, indicates that the *VNN1* gene may promote triglyceride deposition by down-regulating the expression of lipoxinA4. Additionally, the co-analysis of KEGG pathways revealed that differentially expressed genes such as *PPP2R2C*, *IGF1*, *VNN1*, *TNNT2* and *MAPK10* were associated with oxidized lipids, carnitine and ceramide. Although these genes and lipids were differentially expressed, they were not enriched in the same metabolic pathways, suggesting that they may be regulated through different pathways.

Overall, these findings reveal the potential roles of specific genes and lipids in the regulation of goat meat quality. Further investigation of these candidate genes can provide valuable insights into the genetic factors influencing meat quality in goats.

## 5. Conclusions

This study analyzed the dorsal longissimus muscle tissues of goats of different castration status using a combination of metabolomics and transcriptomics. Among the findings, a total of 279 differential lipids were obtained, and the Ras signaling pathway, MAPK signaling pathway and cAMP signaling pathway were found to be enriched. Genes including *IGF1*, *TNNT2*, *MAPK10*, *PPP2R2C* and *VNN1*, along with lipids including glycerol diglycerides, sphingomyelins and lipoxins, were shown to be involved in these pathways. It was concluded that castration can affect the expression of specific genes and lipids that play a role in the regulation of meat quality in goats.

## Figures and Tables

**Table 1:** Effects of castration on slaughter performance of YDGs.

Items	YDW	YDB
SLW (kg)	38.45 ± 5.16	42.63 ± 10.61
Carcass weight (kg)	18.43 ± 2.01	22.00 ± 5.86
Dressing percentage (%)	48.10 ± 2.36	51.45 ± 2.29
Meat weight (kg)	14.15 ± 2.02	17.20 ± 6.08
Meat yield (%)	36.78 ± 1.14	39.58 ± 4.32
LEA (cm^2^)	13.95 ± 1.61	15.45 ± 1.76
GR (mm)	5.25 ± 0.81	6.23 ± 1.74
Backfat thickness (mm)	0.10 ± 0.03	0.10 ± 0.02

Note: SLW = slaughter live weight; LEA = loin eye area; GR = grade rule.

**Table 2:** Effects of castration on amino acid content of YDGs.

Items	YDW	YDB
Asp (%)	2.18 ± 0.14	2.29 ± 0.24
Thr (%)	0.85 ± 0.06	0.90 ± 0.06
Ser (%)	0.78 ± 0.06	0.82 ± 0.06
Glu (%)	3.37 ± 0.23	3.48 ± 0.28
Gly (%)	0.92 ± 0.09	0.93 ± 0.05
Ala (%)	1.29 ± 0.09	1.32 ± 0.07
Val (%)	1.09 ± 0.07	1.14 ± 0.09
Met (%)	0.62 ± 0.06	0.67 ± 0.09
Lle (%)	1.02 ± 0.07	1.10 ± 0.11
Leu (%)	1.89 ± 0.15	1.99 ± 0.24
Tyr (%)	0.76 ± 0.05	0.81 ± 0.08
Phe (%)	0.84 ± 0.03	0.89 ± 0.11
His (%)	0.76 ± 0.15	0.85 ± 0.04
Lys (%)	1.82 ± 0.14	1.93 ± 0.13
Arg (%)	1.33 ± 0.07	1.39 ± 0.14
Pro (%)	0.50 ± 0.04	0.47 ± 0.09

**Table 3:** Effects of castration on meat quality of YDGs.

Items	YDW	YDB
L*	26.89 ± 5.00	29.51 ± 8.97
a*	17.28 ± 2.63	8.80 ± 2.92 *
b*	9.67 ± 0.86	8.54 ± 2.59
PH	6.77 ± 0.35	6.23 ± 0.35
Cooking loss (%)	1.14 ± 0.15	2.09 ± 0.58 *
Shear force (N)	91.33 ± 12.75	85.89 ± 13.99
IMF (%)	2.36 ± 0.82	1.47 ± 0.54
Water (g/100 g)	68.45 ± 2.26	71.95 ± 2.90

Note: L* = lightness; a* = redness; b* = yellowness; * means in the same row with different superscripts shows significant difference (*p* < 0.05).

**Table 4:** Effects of castration on fatty acid contents of YDGs.

Fatty Acids (%)	YDW	YDB
Myristic acid	2.00 ± 0.09	1.98 ± 0.33
Pentadecanoic acid	0.66 ± 0.10	0.15 ± 0.30 *
Palmitic acid	23.90 ± 0.78	24.53 ± 0.97
Heptadecanoic acid	1.67 ± 0.27	0.63 ± 0.74
Stearic acid	25.13 ± 3.62	23.78 ± 4.63
Trans-9-octadecanecarboxylic acid	2.38 ± 0.21	3.64 ± 0.26 *
Cis-9-octadecenoic acid	39.48 ± 3.25	39.00 ± 3.90
Linoleic acid	2.07 ± 0.14	4.52 ± 1.24 *
Cis-11-eicosenoic acid	0.95 ± 0.16	ND
Saturated fatty acid	53.35 ± 3.16	51.05 ± 4.55
Monounsaturated fatty acid	43.73 ± 3.30	43.20 ± 4.31
Polyunsaturated fatty acid	2.89 ± 0.45	5.54 ± 1.94 *

Note: ND = not detected; * means in the same row with different superscripts shows significant difference (*p* < 0.05).

## Data Availability

Data are contained within the article and Supplementary Materials.

## Supplementary Materials

The following supporting information can be downloaded at: https://www.mdpi.com/article/10.3390/genes15010043/s1. Table S1: qPCR Primers List. Table S2: Sequencing Quality Control. Table S3: SCMs in YDB and YDW respect. Table S4: PCA data analysis in YDB and YDW. Table S5: KEGG pathway analysis in SCMs. Table S6: DEGs in YDB and YDW respect. Table S7: GO enrichment in YDB and YDW respect. Table S8: Pathways intergarate genes and metabolisms in YDB and YDW.
